# Sorafenib in Molecularly Selected Cancer Patients: Final Analysis of the MOST-Plus Sorafenib Cohort

**DOI:** 10.3390/cancers15133441

**Published:** 2023-06-30

**Authors:** Olivier Trédan, Maud Toulmonde, Christophe Le Tourneau, Laure Montane, Antoine Italiano, Isabelle Ray-Coquard, Christelle De La Fouchardière, François Bertucci, Anthony Gonçalves, Carlos Gomez-Roca, Benoit You, Valéry Attignon, Sandrine Boyault, Philippe A. Cassier, Armelle Dufresne, Séverine Tabone-Eglinger, Alain Viari, Emilie Sohier, Maud Kamal, Gwenaël Garin, Jean-Yves Blay, David Pérol

**Affiliations:** 1 Medical Oncology Department, Centre Léon Bérard, 69008 Lyon, France; isabelle.ray-coquard@lyon.unicancer.fr (I.R.-C.); jean-yves.blay@lyon.unicancer.fr (J.-Y.B.); 2 Inserm U1052, Centre de Recherche en Cancérologie de Lyon, F-69000 Lyon, France; 3 Medical Oncology Department, Institut Bergonié, 33076 Bordeaux, France; m.toulmonde@bordeaux.unicancer.fr (M.T.); a.italiano@bordeaux.unicancer.fr (A.I.); 4 Department of Drug Development and Innovation (D3i), Institut Curie, Paris-Saclay University, 75005 Paris, France; christophe.letourneau@curie.fr (C.L.T.); maud.kamal@curie.fr (M.K.); 5 Department of Clinical Research and Innovation, Léon Bérard Cancer Center, 69008 Lyon, Francegwenaelle.garin@lyon.unicancer.fr (G.G.);; 6 Department of Medical Oncology, Institut Paoli-Calmettes, 13009 Marseille, France; 7 Medical Oncology Department, Institute Universitaire de Cancérologie de Toulouse, 31037 Toulouse, France; gomez-roca.carlos@iuct-oncopole.fr; 8 Department of Medical Oncology, Lyon Sud Hospital Center, CITOHL, Institute of Cancerology, Hospices Civils de Lyon (IC-HCL), 69495 Lyon, France; benoit.you@chu-lyon.fr; 9 Genomic Platform, Léon Bérard Cancer Center, 69008 Lyon, France; 10 Biological Ressources Center, Léon Bérard Cancer Center, 69008 Lyon, France; 11 Gilles Thomas Bioinformatic Platform, Léon Bérard Cancer Center, 69008 Lyon, France

**Keywords:** personalized medicine, biologically driven trial, sorafenib, randomized discontinuation design

## Abstract

**Simple Summary:**

Using a randomized discontinuation design, sorafenib was tested on patients with advanced/metastatic solid tumors previously treated by standard treatments and harboring sorafenib-targeted genes. Patients with stable disease after 12 weeks of sorafenib induction treatment were randomized equally between continuation or interruption of sorafenib. Continuing sorafenib when stable disease is achieved, after a 12-week induction treatment, improves progression free rate compared to interruption. Sorafenib has tumor-agnostic efficacy in patients with tumors harboring genomic alterations in PDGFRA/B, VEGF-Rs, Flt-3, KIT, FGFR1 or the RAF/MEK/ERK pathway.

**Abstract:**

Background: MOST-plus is a multicenter, randomized, open-label, adaptive Phase II trial evaluating the clinical benefit of targeted treatments matched to molecular alteration in advanced/metastatic solid tumors. Sorafenib was tested on patients with tumors harboring sorafenib-targeted genes. Methods: The MOST-plus trial used a randomized discontinuation design. After 12 weeks of sorafenib (400 mg, po BID), patients with progressive disease discontinued study, patients with objective response were proposed to continue sorafenib, whereas patients with stable disease (SD) were randomly assigned (1:1) to the maintenance or interruption of treatment. The primary endpoint was RECIST version 1.1 progression-free rate at 16 weeks after randomization (PFR-16w). Secondary endpoints included progression-free survival (PFS), overall survival (OS), and toxicity. Statistical analyses used a sequential Bayesian approach with interim efficacy analyses. The enrolment could be stopped in the case of a 95% probability for the estimated PFR-16w to be higher in the maintenance than in the interruption arm (NCT02029001). Results: 151 patients were included, of whom 35 had SD at 12 weeks of Sorafenib. For the 35 patients with SD on sorafenib, the PFR-16w was 65% [95% credibility interval 43.4–83.7] in the continuation arm and 25% [7.8–48.1] in the interruption arm. Median PFS and OS were improved in the maintenance versus the interruption arm (mPFS: 5.6 [95%CI 1.97–6.77] months versus 2.0 [95%CI 1.61–3.91] months (*p* = 0.0231) and mOS: 14.3 [95%CI 8.9–23.8] versus 8.0 months [95%CI 3.5–15.2] (*p* = 0.0857)). Conclusion: Sorafenib showed activity in progressive patients with solid tumors harboring somatic genomic alterations in sorafenib-targeted genes. Continuing sorafenib when SD is achieved improves PFR compared to interruption.

## 1. Introduction

The emergence of next-generation sequencing [NGS] led to the identification of molecular alterations in genes involved in tumor progression in a variety of cancers [1]. Molecular profiling efforts from the International Cancer Genomics Consortium (ICGC), or The Cancer Genome Atlas (TCGA) have shown the multiplicity, complexity, diversity, and heterogeneity of genomic alterations within cancer types [2]. Several genomic-driven clinical trials have allowed researchers to assess the therapeutic value of matching drugs with specific tumor characteristics [3,4,5,6,7,8,9,10,11]. It remains questionable whether disease stabilization results from drug efficacy or from intrinsic low-proliferating tumors independently of targeted agent efficacy [12].

MOST-plus is a multicenter, randomized, open-label, adaptive platform phase II [13] aiming to assess the clinical benefit of continuing a biomarker-allocated treatment in advanced/metastatic solid tumors using a randomized discontinuation design (RDD [14]). This genomic-driven study evaluated seven targeted therapies: nilotinib, everolimus, sorafenib, lapatinib, pazopanib, olaparib, and durvalumab + tremelimumab. Each treatment cohort is conducted with common procedures for quality control and reporting. However, MOST Plus is designed as a master protocol with independent cohort of treatments that are analyzed separately according to enrolment rate. The publication plan is based on independent publication for each cohort for the sake of clarity. In this manuscript, we present the final analysis of the sorafenib cohort.

The oral multi-targeted tyrosine kinase inhibitor (TKI) sorafenib inhibits platelet-derived growth factor receptors (PDGFR), vascular endothelial growth factor (VEGF-R), Fms-like tyrosine kinase-3 (Flt-3), tyrosine-protein kinase KIT (also known as CD117 or mast/stem cell growth factor receptor (SCFR)), RET (rearranged during transfection) [15] and fibroblast growth factor receptors FGFR1. Sorafenib inhibits tumor growth and angiogenesis through targeting both the RAF/MEK/ERK pathway and receptor tyrosine kinases [16]. Sorafenib treatment results in a cytostatic rather than a cytotoxic effect; thus, the expected primary clinical benefit is disease stabilization rather than objective disease shrinkage. Sorafenib is approved for the treatment of unresectable hepatocellular carcinoma, advanced renal cell carcinoma, and locally advanced or metastatic, differentiated thyroid carcinoma [17,18,19]. 

The hypothesis of the MOST-plus trial is that continuing a targeted therapy such as sorafenib in patients with matched genomic alterations and stable disease after a 12-week induction period could improve clinical outcomes.

## 2. Materials and Methods

MOST-plus (My Own Specific Treatment, NCT02029001) is a multicenter, randomized, open-label, genomic-driven, adaptive phase II platform trial. The trial was conducted according to Good Clinical Practice guidelines, the Declaration of Helsinki, and relevant French and European laws and directives. All patients provided written informed consent. 

### 2.1. Study Population

Eligible patients were 18 years of age or older, with histologically confirmed advanced/metastatic solid tumors (any type) treated by at least one line of prior chemotherapy, and harboring at least one of the following molecular alterations according to local assessment: mutations or amplification/translocation in VEGFR1-3, PDGFRB, FLT3, BRAF (excluding V600E), CRAF, HRAS, KRAS, or RET, and/or cognate ligands. Patient genomic profiles were reviewed by a centralized virtual Molecular Tumor Board before patient enrolment. Other key eligibility criteria included adequate performance status according to Eastern Cooperative Oncology Group (ECOG), performance status of 0 to 2, presence of at least one measurable lesion as per the Response Evaluation Criteria In Solid Tumors, version 1.1 (RECIST 1.1 [20]), documented disease progression at inclusion, and normal organ and bone marrow functions confirmed within 7 days before sorafenib initiation. 

Randomization was stratified according to Eastern Cooperative Oncology Group (ECOG) performance status (0–1 versus 2) (See the study protocol in Supplementary Material for further details).

### 2.2. Study Procedures and Assessments

All eligible patients were initially treated with biomarker-allocated treatment for 12 weeks (sorafenib: 400 mg, twice daily, per os). According to RDD, at the end of this induction period, patients with an objective response (OR) continue on therapy; patients with progressive disease (PD) permanently discontinue therapy, whereas patients with stable disease (SD) were randomly allocated (1:1) to either continue (maintenance arm) or discontinue (interruption arm) the allocated therapy. Randomization was carried out using a web-based system and was stratified (permuted block) by performance status (0 vs. 1–2). The biostatiscian generated the randomization sequence; patients were enrolled by study coordinator into eCRF and were randomized depending on tumor assessment following induction period treatment. Patients in the interruption arm could reinitiate targeted therapy upon documented disease progression following treatment interruption.

Protocol-defined dose modifications, including interruptions and dose reductions, were used to manage adverse events (AEs) according to sorafenib summary of product characteristics. AE were graded according to the Common Terminology Criteria for Adverse Events (CTCAE -version 4.03). Disease assessments with computed tomography or magnetic resonance imaging were performed at baseline, at week 12 (W12) and then every 8 weeks until disease progression, death or withdrawal. Tumor responses were determined by the investigator according to RECIST V1.1 (See the study protocol in Supplementary Material for further details). 

### 2.3. Endpoints and Statistics

The primary endpoint was the proportion of randomized patients remaining progression-free at 16 weeks after randomization (PFR-16w) according to RECIST 1.1. Secondary endpoints included overall response rate, duration of response, progression-free survival (PFS), overall survival (OS), and safety. 

The study used a Bayesian adaptive phase II design, allowing updating knowledge gradually rather than restricting revisions in a trial design with fixed sample sizes [21,22]. The analysis of the primary endpoint (PFR-16w) was carried out sequentially, with interim analyses planned after a 16-week follow-up for the first 20 randomized patients, then every 10 randomized patients. The probability of success (PFR-16w) was estimated from a beta-binomial model. In the absence of strong ideas about the non-progression rate, a non-informative prior distribution beta (1,1) was considered. At each interim analysis, the trial could be stopped if there was a high posterior probability (≥95%) that the PFR-16w was higher in the maintenance than in the interruption arm. At the end of the trial, if no stopping rule occurred, the maintenance arm was considered superior if the posterior probability for the PFR-16w to be higher in the maintenance arm was at least 90%. Mean PFR-16w estimated by the Bayesian method were described in each arm along with the associated 95% credibility intervals (CrI) (precision of the Bayesian estimation). 

Maximum sample size was set at 50 randomized patients for each treatment cohort.

The median follow-up was calculated using the reverse Kaplan–Meier method. OS and PFS were estimated using the Kaplan–Meier method and described in terms of median along with associated 2-sided 95% confidence interval (CI) calculated by Brookmeyer and Crowley technique. Data for patients who were event-free at the time of analysis were censored at the date of the last follow-up for OS and at the time of last assessment for PFS. Patients randomized while they had disease progression were censored at the time of randomization for PFS analysis. For exploratory purposes, OS and PFS distributions were compared between the two randomization arms using a log-rank test stratified on ECOG Performance status at randomization.

Quantitative variables were described using the median and IQR. Qualitative variables were described using frequency and 95%CI. Efficacy data are presented according to disease status at the end of the induction period (i.e., at W12) for evaluable patients treated during at least one cycle. All patients having received at least one cycle of sorafenib were assessed for safety. Evaluable patients for primary endpoint include all patients who received at least one cycle of sorafenib or discontinued treatment earlier for a reason other than PD, death or related toxicity. Based on the ITT principle, PFS and OS analysis were performed including all randomly assigned patients. Data cutoff was February 27, 2020. Statistical analyses were performed using SAS software (version 9.4, SAS Institute Inc., Cary, NC, USA)

## 3. Results

### 3.1. Patient Characteristics and Trial Profile

**Figure 1:**
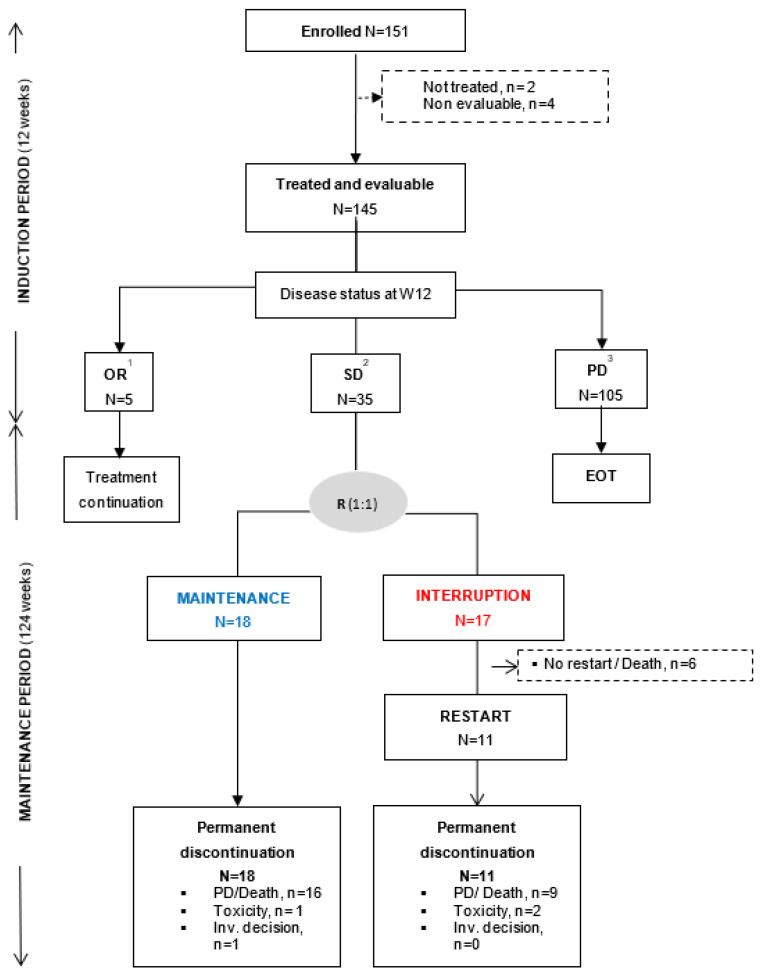
**Trial profile.** Following an induction period of 12 weeks, 145 treated and evaluable patients were categorized according to disease status: patients with objective response (OR, n = 5) continued sorafenib; patients with stable disease (SD, n = 35) were randomized (R) with a 1:1 ratio to the maintenance (blue) or interruption (red) arm; and patients with progressive disease (PD, n = 105) permanently discontinued sorafenib. Randomization was stratified according to ECOG PS: 0−1 versus 2 at randomization. Non-evaluable patients were defined as patients with less than one cycle of treatment due to a reason other than disease progression, sorafenib-related toxicities, or death. ^1^ All patients with OR at W12 have permanently discontinued sorafenib due to PD at time of database cutoff. ^2^ Five patients in the interruption arm were randomized without documented SD: PD (n = 3), PR (n = 1), and NE (n = 1), and one patient was randomized in maintenance arm despite >28 days sorafenib temporary discontinuation before randomization. ^3^ This subgroup also includes patients with sorafenib permanent discontinuation before or at W12 due to toxicity (n = 34), investigator or patient decision (n = 14), death (n = 10), another reason (n = 1 with abnormal ECG at time of randomization).

From April 2014 to May 2018, 151 patients with advanced/metastatic solid tumors were enrolled in the sorafenib cohort. Among them, two patients were not treated and four patients were treated but did not receive the full first cycle of sorafenib due to patient decision. These six patients were considered as non-evaluable for efficacy endpoints. All analyses were performed on the 145 patients having received at least one complete cycle of sorafenib or who discontinued earlier for a reason other than PD, death or sorafenib-related toxicity (Figure 1).

**Figure 2:**
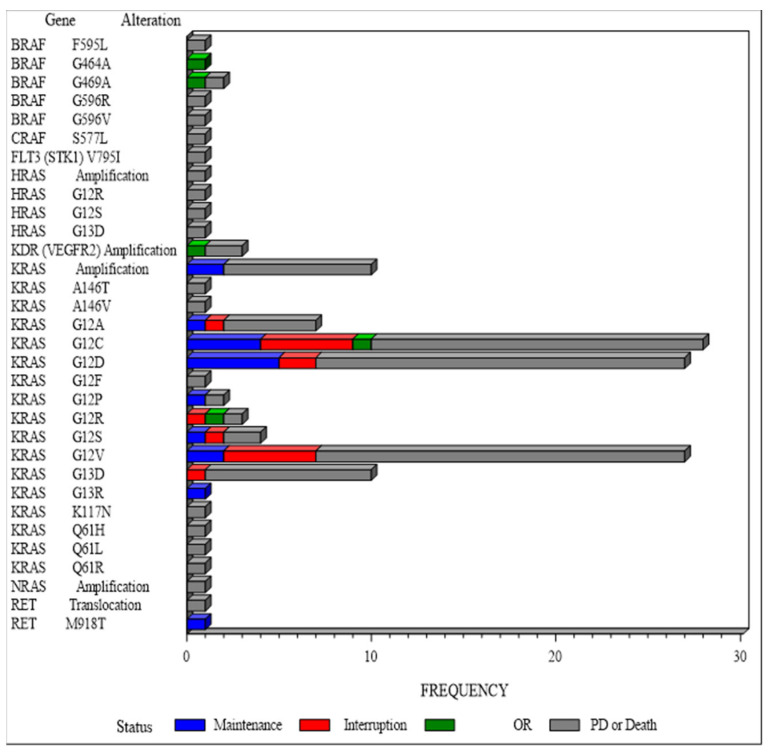
Detailed graph of molecular alteration having led to sorafenib recommendation. Molecular alterations per gene name and type of alteration according to disease status at Week 12.

Baseline characteristics are presented in Table 1. The most common (≥5%) primary tumor sites were lung (26.2%), colorectal (25.5%), gynecological (15.2%), and pancreas (12.4%). Almost all patients had metastatic disease at inclusion (94%) and were heavily pre-treated with a median number of prior treatment lines of 3 [1,2,3,4,5,6,7,8,9,10,11]. Chemotherapy was the main treatment type (62.1%) administered before inclusion. The molecular alterations that allowed patient inclusion were mainly KRAS hot-spot mutations (Figure 2). According to ESCAT classification [22], the majority of molecular alterations were ESCAT IV (71%) with only one patient with ESCAT I molecular alteration (a lung adenocarcinoma with RET translocation) and 22 patients with ESCAT II (14.5%) molecular alteration (mainly pancreatic cancer with KRAS host spot mutation).

### 3.2. Primary Efficacy Endpoint 

**Figure 3:**
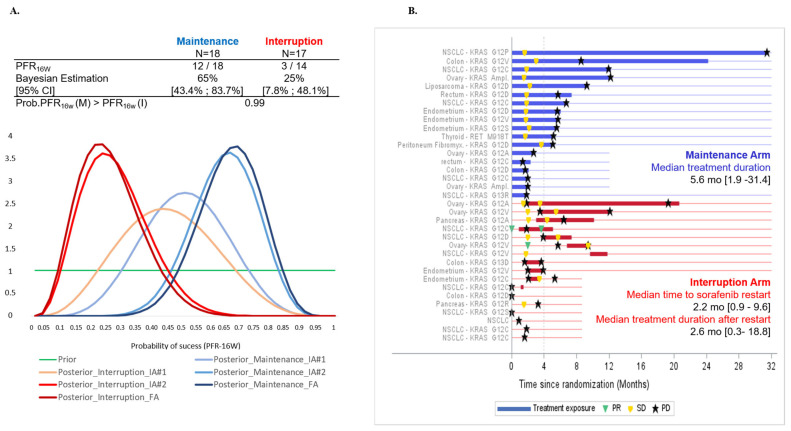
**Progression Free rate at 16 weeks post-randomisation (PFR-16W), duration of treatment and best response from randomization.** (**A**). Bayesian estimates of the probability distribution of being progression-free (success) at 16 weeks after randomization. Prior and posterior density functions of the probability of success were updated after each successive interim analysis. Success was defined as being progression-free at 16 weeks. (**B**). Duration of treatment and best response from randomization. Primary tumor site and molecular alterations having led to inclusion in the sorafenib cohort are listed for each patient. In the interruption arm (red lines), patients were proposed to reinitiate sorafenib in case of PD (black star). One patient was enrolled without molecular alteration and randomized in the interruption arm.

At the end of the induction period: 5 (3.5%) patients with OR (5 PR) continued sorafenib, 105 (72.4%) discontinued sorafenib at/or before W12 mainly due to PD or death or unacceptable toxicity, whereas 35 (24.1%) patients with SD were randomized (1:1) to the maintenance (N = 18) or interruption (N = 17) arms (Figure 2). In the interruption arm, sorafenib was re-introduced after treatment interruption and PD in 11 out of 17 patients (64.7%). One patient was randomized with a PR and discontinued the study one week after randomization. Among the 35 randomized patients, three patients with investigator-assessed SD at W12 were finally documented with PD at W12. They were randomly assigned to the interruption arm instead of discontinuing treatment and were therefore censored at the date of randomization for PFS analysis. At the time of final analysis, all patients had discontinued sorafenib treatment, mainly due to PD (77.9%, Figure 1). At the second interim analysis, PFR-16w was 65% [95%CrI 43.4–83.7] for the maintenance arm and 25% [CrI 95%: 7.8–48] for the interruption arm (Figure 3A). With a probability that the maintenance arm was superior to interruption arm of 99%, the stopping rule applied and the recruitment to the sorafenib cohort prematurely stopped.

### 3.3. Secondary Efficacy Endpoints 

**Figure 4:**
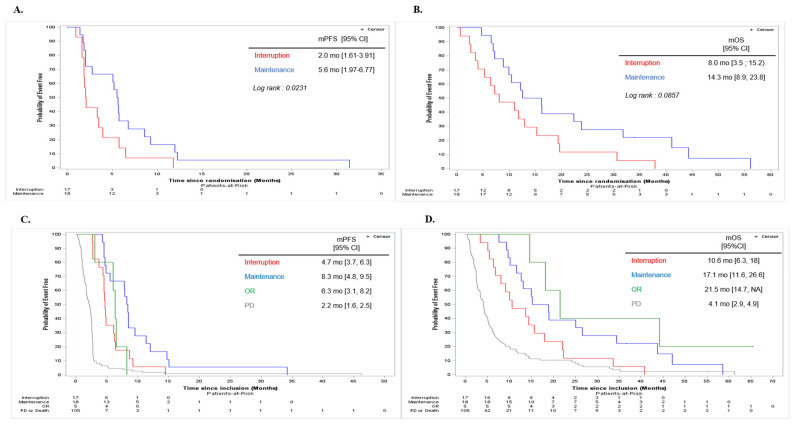
**Kaplan Meier plots for progression free survival and overall survival** (**A**) PFS from randomization. (**B**) OS from randomization. (**C**) PFS from inclusion. (**D**) OS from inclusion.

Tumor response and median treatment duration post-randomization are presented in Figure 3B. In the maintenance arm, there was no objective response and the median duration of treatment post-randomization was 5.6 [1.9−31.4] months. Among the 11 patients of the interruption arm in whom sorafenib was re-started following PD, 2 (18.2%) patients reached PR after sorafenib reintroduction. Median time to treatment reinitiation was 2.2 [0.9–9.6] months and the median duration of sorafenib treatment following restart was 2.6 [0.3–18.8] months. The median PFS was significantly higher in the maintenance arm than in the interruption arm (5.6 [95%CI 1.97–6.77] months and 2.0 [95%CI 1.61–3.91] months, respectively [log-rank *p* = 0.0231, Figure 4A]). Median OS from randomization was 14.3 [95%CI 8.9–23.8] months in the maintenance arm and 8.0 months [95%CI 3.5–15.2] in the interruption arm (log-rank *p* = 0.0857, Figure 4B). Median PFS following sorafenib reinitiation was 2.8 [95%CI 1.2–4.1] months. Median OS was 11.8 [95%CI 6.4–19] months after sorafenib reinitiation, versus 3.8 [95%CI 0.6–19.6] months in patients who did not reinitiate sorafenib. Median PFS from inclusion (N = 145) was 4.7 [3.7–6.3] months in the interruption arm and 8.3 [4.8–9.5] months in the maintenance arm, 6.3 [3.1–8.2] months in patients with OR, and 2.2 [1.6–2.5] months in patients with PD at the end of the induction period (Figure 4C). Median OS from inclusion was 10.6 [6.3–18] months in the interruption arm, 17.1 months [11.6–26.6] in the maintenance arm, 21.5 [14.7–NR] months in patients with OR, and 4.1 [2.9−4.9] months in patients with PD at the end of the induction period. 

### 3.4. Safety Endpoints

Almost all treated patients (87%) experienced at least one sorafenib-related AE including 46% with at least one grade ≥ 3 sorafenib-related AE (Table 2). Consistently with the known safety profile of sorafenib, the most common (≥5% in overall population) grade ≥ 3 sorafenib-related AE were vomiting (6.2%), fatigue (7.6%), hand andfoot syndrome (6.2%), and hypertension (12.4%). A total of three unexpected deaths was reported by investigators: a fatal dyspnea with various hypothesis of death pulmonary embolism, arrhythmia or stroke all possibly related to sorafenib, (N = 1), a cardio-respiratory arrest of unknown etiology (N = 1), and an acute coronary syndrome in a patient with typical ventriculography of Tako-Tsubo (N = 1). 

## 4. Discussion

This multicenter, randomized, open-label phase II demonstrates that maintaining sorafenib treatment for molecularly selected patients, when the disease is not progressing during an induction period, improves PFS. Based on the previous MD Anderson Cancer Center experience, it was initially expected that approximately 65% of enrolled patients would experience a SD after 12 weeks of treatment [23]. In our sorafenib cohort, the randomization rate at 12 weeks was lower than expected (i.e., 24%). However, this was consistent with the data obtained by Ratain et al. [12] in a RDD phase II trial reporting a non-progression rate of 32% following an induction period of 12 weeks with sorafenib in metastatic renal cell carcinoma. In agreement with our results, the authors showed that maintaining sorafenib significantly improved the rate of non-progression compared to the placebo group (PFR-24w: 50% versus 18% [*p* = 0.0077]). Nevertheless, the efficacy of the sorafenib maintenance treatment may depend on the histology of the primary tumor. Indeed, during the course of our trial, the low percentage of colorectal cancer patients achieving SD after the induction period, led to the interruption of the sorafenib recommendation for patients with colorectal cancer, as supported by Samalin et al. [24]. 

One weakness of our trial is that the molecular screening program in the MOST-plus trial was not centralized; each institution performed its own genomic analysis, thus leading to heterogeneity of NGS panels used and missing data about other relevant molecular alterations including, potentially, resistance mutation. However, a centralized molecular tumor board reviewed the sorafenib treatment indication before enrollment. The vast majority of our patients (87%) had hot-spot KRAS-mutated tumors, including 28% KRASG12C (OR group: N = 1/5, SD group: N = 9/35, PD group: N = 18/105 [Figure 2]). Analysis of this cohort did not allow us to identify an association between a given molecular alteration and outcomes. Results from precision medicine clinical trials are often biased by the fact that tumor profiling before inclusion frequently used heterogeneous tumor samples and different sequencing technologies. Furthermore, the predictive value of known mutations for targeted treatment depends on the tumor type and the presence of other relevant, potentially resistant, alterations. In line with this, several studies have shown that the activation of the MAP kinase pathway downstream of a RAS mutation is mutation- and tissue-specific [25]. Several mechanisms were described as being involved in the acquired resistance to sorafenib, such as reactivation of wild-type *KRAS*, crosstalk between PI3K/Akt and JAK-STAT pathways, or the activation of hypoxia-inducible pathways and epithelial-mesenchymal transition [26]. This was not analyzed in our study. 

Sorafenib is a multi-target kinase inhibitor, not selective for *KRAS* mutation [15]. Several drugs that more specifically target components of the MAP kinase pathway have been developed, including allele-specified. Sotorasib is a specific and irreversible inhibitor of KRASG12C that covalently traps KRASG12C in the inactive state, thus inhibiting KRAS oncogenic signaling [27]. A recent phase II has demonstrated that sotorasib as single agent had significant clinical activity in KRASG12C non-small-cell lung cancer, which has led to its recent approval by the Food and Drug administration (N = 126 patients, with objective response rate of 37.1% [95%CI 28.6–46.2] and a disease control rate of 80.6% [95%CI 72.6–87.2] [28]. The NEXIRI trial suggests that the efficacy of sorafenib may also depend on histological tumor type [24]. This is also supported by other studies on MAP kinase pathway inhibitors in CRC, though differences in *KRAS* alleles may also be at play [25,29]. 

One strength of our trial is the use of an adaptive approach. The Bayesian method adopted in the MOST-plus platform trial was designed to quickly modify the course of each ongoing cohort through regular updating in information during the study, and more specifically to allow early termination of uninteresting cohorts. To be effective and deliver the required results, such an approach requires a predefined number of steps with regards to the planning of the trial. First, a reasonable hypothesis about the non-progression rate for each cohort needs to be determined with clinicians to reflect a clinically relevant desirable outcome. A non-informative prior distribution should only be considered in the absence of a strong idea about the probability of success, which was the case in the sorafenib cohort of MOST-plus, but should remain as much as possible an exception. Second, the adaptive algorithms must be pre-specified with precise stopping rules in order to minimize operational biases. Finally, the requirement for computer-based simulations requires more planning time than experimental designs based on a frequentist methodology. The second methodological choice implemented in the MOST-plus trial was a randomization discontinuation design, RDD, which allows the enrichment of study population with selected patients following an initial induction treatment period and the conduct of a controlled trial with reduced sample size and limited use of placebo (compared to upfront randomized trials). In addition, all patients in RDD initially receive the targeted agent (during the induction period), which partially explains the few partial responses observed and prolonged survival in this sub-population. Although RDD design presents some advantages, it often requires enrolling more patients to reach the expected sample size for the randomized part of the trial, leading to increased study duration and costs. Such a design may not be the best choice to rapidly select a relevant molecular subtype for a given targeted therapy. A new adaptive personalized medicine program without RDD is currently ongoing in our institution to evaluate several targeted therapies (MegaMOST trial, NCT04116541). The rhythm of interim analysis is much faster than in the MOST Plus trial and this should allow to more rapidly communicate final study results.

Future plans for a precision medicine program could include the use of (i) machine-learning methods to optimize treatment algorithms to allow better prediction of response to targeted therapy, and (ii) longitudinal molecular screening, with repeated tumor and blood sampling, that could potentially lead to more relevant targeted therapy recommendation. Indeed, during the course of disease and/or of treatment, tumors become more heterogeneous and include a collection of cells harboring distinct molecular signatures with differential levels of sensitivity to treatment. Assessment of tumor heterogeneity and plasticity are essential for the development of effective therapies. Longitudinal analysis of biopsy samples is of considerable interest to assess the complex clonal architecture of cancers and potentially adapt cancer treatment to tumor profile/characteristics over time. In this context, the profiling of circulating tumor DNA using non-invasive liquid biopsies is also an interesting approach to assess cancer evolution and clonal heterogeneity. A longitudinal screening program with tumor and liquid biopsies is currently ongoing in our institution (PLANET trial, NCT05099068).

## 5. Conclusions

In conclusion, the MOST-plus sorafenib cohort validates that agents targeting Ras/Raf/MEK/ERK signaling pathway may achieve prolonged tumor control. In such a situation, the continuation of targeted therapy should be the rule. Novel KRAS-specific inhibitors should be further explored in non-histology-specific tumors bearing specific genomic alterations.

## Figures and Tables

**Table 1:** Patient demographics and baseline characteristics.

	Disease Status at End of Induction Period								Total	
	OR		PD		SD					
					Maintenance		Interruption			
	n = 5		n = 105		n = 18		n = 17		n = 145	
**Age (years)**										
Median (IQR)	66.0 (61.0−70.0)		63.0 (56.0−68.0)		60.0 (55.0−65.0)		65.0 (57.0−68.0)		63.0 (56.0−68.0)	
**Sex**										
M	3	(60.0)	49	(46.7%)	8	(44.4%)	6	(35.3%)	66	(45.5%)
F	2	(40.0)	56	(53.3%)	10	(55.6%)	11	(64.7%)	79	(54.5%)
**PS ECOG, n (%)**										
0	3	(60.0)	28	(26.7%)	10	(55.6%)	6	(35.3%)	47	(32.4%)
1	2	(40.0)	63	(60.0%)	7	(38.9%)	9	(52.9%)	81	(55.9%)
2	0	(0.0%)	14	(13.3%)	1	(5.6%)	2	(11.8%)	17	(11.7%)
**Main primary tumor site (≥10% in at least one subgroup), n (%)**										
CRC	0	(0.0%)	31	(29.5%)	4	(22.2%)	2	(11.8%)	37	(25.5%)
H & N	1	(20.0%)	0	(0.0%)	0	(0.0%)	0	(0.0%)	1	(0.7%)
Retroperitoneal	0	(0.0%)	0	(0.0%)	2	(11.1%)	0	(0.0%)	2	(1.4%)
Gynecological.	0	(0.0%)	18	(17.1%)	6	(33.3%)	5	(29.4%)	29	(20.0%)
NSCLC	3	(60.0%)	22	(21.0%)	5	(27.8%)	8	(47.1%)	38	(26.2%)
Prostate	1	(20.0%)	0	(0.0%)	0	(0.0%)	0	(0.0%)	1	(0.7%)
Pancreas	0	(0.0%)	16	(15.2%)	0	(0.0%)	2	(11.8%)	18	(12.4%)
**Prior number of lines in advanced/metastatic stage**										
Median (IQR)	5.0 (3.0−6.0)		3.0 (2.0−4.0)		2.5 (2.0−4.0)		3.0 (2.0−3.0)		3.0 (2.0−4.0)	
1 L or 2 L	1 (20%)		50 (47.6%)		9 (50.0%)		8 (47.1%)		68 (46.9%)	
3 L to 5 L	2 (40%)		43 (41.0%)		8 (44.4%)		8 (47.1%)		61 (42.1%)	
≥6 L	2 (40%)		12 (11.4%)		1 (5.6%)		1 (5.8%)		16 (11.0%)	
**Type of prior line before inclusion, n (%)**										
CT	3	(60.0)	68	(64.8%)	7	(38.9%)	12	(70.6%)	90	(62.1%)
CT + Anti-angiogenic	0	(0.0%)	27	(25.7%)	5	(27.8%)	2	(11.8%)	34	(23.4%)
Targeted therapy	1	(20%)	7	(6.6%)	5	(27.8%)	2	(11.8%)	15	(10.3%)
Immunotherapy	0	(0.0%)	2	(1.9%)	1	(5.6%)	0	(0.0%)	3	(2.1%)
Hormonotherapy	0	(0.0%)	1	(1.0%)	0	(0.0%)	1	(5.9%)	2	(1.4%)
**Best response to prior line according to RECIST V1.1, n (%)**										
CR	0	(0.0%)	2	(1.9%)	0	(0.0%)	0	(0.0%)	2	(1.4%)
PR	1	(20.0)	8	(7.6%)	0	(0.0%)	2	(11.8%)	11	(7.6%)
SD	0	(0.0%)	36	(34.3%)	7	(38.9%)	12	(70.6%)	55	(37.9%)
PD	2	(40.0)	56	(53.3%)	10	(55.6%)	2	(11.8%)	70	(48.3%)
NE	1	(20.0)	3	(2.9%)	1	(5.6%)	1	(5.9%)	6	(4.1%)

In grey: patients randomized at the end of induction period. OR: objective response, PD: progressive disease; SD: Stable disease, CR: complete response, PR: partial response, NE: non-evaluable, CRC: colorectal, H&N: Head and neck squamous cell carcinoma, NSCLC: non-small cell lung cancer; L: lines of treatment. Data are n (%), and median.

**Table 2:** Adverse events summary.

	Disease Status at End of Induction Period								TOTAL	
	OR		PD		SD					
					Maintenance		Interruption			
	n = 5		n = 105		n = 18		n = 17		n = 145	
**Number of patients with at least, n (%)**										
One AE (all grades)	5	(100.0%)	105	(100.0%)	18	(100.0%)	17	(100.0%)	145	(100.0%)
One sorafenib-related AE (all grades)	5	(100.0%)	86	(81.9%)	18	(100.0%)	17	(100.0%)	126	(86.9%)
One Grade ≥ 3 AE	1	(20.0%)	84	(80.0%)	13	(72.2%)	14	(82.4%)	112	(77.2%)
One Grade ≥ 3 sorafenib-related AE	1	(20.0%)	46	(43.8%)	12	(66.7%)	8	(47.1%)	67	(46.2%)
One related SAE	0	(0.0%)	33	(31.4%)	6	(33.3%)	4	(23.5%)	43	(29.7%)
One SUSAR	0	(0.0%)	10	(9.5%)	0	(0.0%)	1	(5.9%)	11	(7.6%)
**Main (≥ 5%) Grade ≥ 3 related AE, n (%)**										
Abdominal pain	0	(0.0%)	1	(1.0%)	1	(5.6%)	0	(0.0%)	2	(1.4%)
Intestinal perforation	0	(0.0%)	0	(0.0%)	1	(5.6%)	0	(0.0%)	1	(0.7%)
Vomiting	0	(0.0%)	4	(3.8%)	5	(27.8%)	0	(0.0%)	9	(6.2%)
Fatigue	0	(0.0%)	9	(8.6%)	2	(11.1%)	0	(0.0%)	11	(7.6%)
QT prolonged	0	(0.0%)	0	(0.0%)	0	(0.0%)	1	(5.9%)	1	(0.7%)
GGT increased	0	(0.0%)	3	(2.9%)	1	(5.6%)	0	(0.0%)	4	(2.8%)
Weight decreased	0	(0.0%)	0	(0.0%)	1	(5.6%)	0	(0.0%)	1	(0.7%)
WBC decreased	0	(0.0%)	0	(0.0%)	0	(0.0%)	1	(5.9%)	1	(0.7%)
Hypocalcemia	0	(0.0%)	0	(0.0%)	1	(5.6%)	0	(0.0%)	1	(0.7%)
Dyspnea	0	(0.0%)	0	(0.0%)	0	(0.0%)	1	(5.9%)	1	(0.7%)
Pulmonary embolism	0	(0.0%)	0	(0.0%)	0	(0.0%)	1	(5.9%)	1	(0.7%)
Hand and Foot syndrome	0	(0.0%)	4	(3.8%)	4	(22.2%)	1	(5.9%)	9	(6.2%)
Rash	0	(0.0%)	2	(1.9%)	0	(0.0%)	1	(5.9%)	3	(2.1%)
Hypertension	1	(20.0%)	10	(9.5%)	3	(16.7%)	4	(23.5%)	18	(12.4%)

AE: adverse event, Gr.: Grade according to NCI-CTCAE v4.03, SAE: Serious adverse event.

## Data Availability

The data presented in this study are available on request from the corresponding author. The data are not publicly available due to legal, ethical and contractual reasons.

## Supplementary Materials

The following supporting information can be downloaded at: https://www.mdpi.com/article/10.3390/cancers15133441/s1, File: Study protocol.
