# Supplementary material for: Sorafenib in Molecularly Selected Cancer Patients: Final Analysis of the MOST-Plus Sorafenib Cohort
Source: Cancers (Basel). 2023 Jun 30;15(13):3441. doi: 10.3390/cancers15133441 (PMC10340237; doi:10.3390/cancers15133441)
Supplement: Supplementary file 1 [file cancers-15-03441-s001.zip › cancers-2421649-supplementary.pdf]

NOTE D'INFORMATION DESTINEE AUX PERSONNES A QUI EST PROPOSEE L'ESSAI CLINIQUE

MOST *Plus*

Etude multicentrique de phase II en deux périodes, randomisée, en ouvert évaluant le bénéfice clinique d'un traitement d'entretien par une thérapie ciblée adaptée aux altérations moléculaires identifiées dans la tumeur pour des patients atteints de tous types de tumeurs solides localement avancées ou métastatiques en progression.

Version 6.0 du 14 juin 2022

Bras de traitement : Durvalumab + Tremelimumab

Promoteur : CENTRE LEON BERARD - 28 rue Laennec, 69373 LYON Cedex 08

Identifiants de la recherche : N° Promoteur : ET12-081 / N°EudraCT (base de données européenne) : 2012-004510-34

Madame, Monsieur,

Le Dr………………………………………………………….. vous propose maintenant de participer à l'essai **MOST *Plus***. Avant de vous décider, il est important que vous compreniez pourquoi cette étude est réalisée et ce qu'elle implique.
Ce document a été conçu pour vous apporter ces informations. Prenez votre temps pour le lire. Vous pouvez poser toutes les questions que vous souhaitez au médecin investigateur, c'est-à-dire l'oncologue qui vous a proposé cet essai clinique. Avant de prendre une décision, vous pouvez également discuter de votre participation avec votre médecin référent ou vos proches. En particulier, il convient d'aborder avec votre médecin cancérologue les différentes alternatives thérapeutiques possibles.

Après avoir lu attentivement ce document et disposé d'un délai de réflexion suffisant :

- Si vous acceptez de participer à cette étude, votre médecin vous demandera de donner votre consentement par écrit en datant et signant le formulaire de consentement de participation ; vous en conserverez un exemplaire.
- Si vous refusez de participer à cette étude, cela n'affectera en rien la qualité des soins qui vous seront proposés.

1. OBJECTIF de L'ETUDE 2
2. LE TRAITEMENT ETUDIE 2
3. DEROULEMENT DE L'ETUDE 3
4. BENEFICES ATTENDUS, CONTRAINTES ET RISQUES PREVISIBLES 6
5. ECHANTILLONS BIOLOGIQUES PRELEVES DANS LE CADRE DE CET ESSAI CLINIQUE 9
6. VOTRE PARTICIPATION A CET ESSAI CLINIQUE : COMPLEMENT D'INFORMATION 9
7. A QUI S'ADRESSER EN CAS DE QUESTIONS OU DE PROBLEME ? 12

1. OBJECTIF DE L'ETUDE

Actuellement, l'option thérapeutique la plus souvent utilisée en cancérologie est le traitement par chimiothérapie, dont la nature varie en fonction du type et de la localisation de la tumeur. Les chimiothérapies sont des médicaments ayant une action sur l'organisme dite « non ciblée », c'est-à-dire qu'ils agissent en détruisant les cellules tumorales, mais aussi certaines cellules saines en cours de division. Cette action non ciblée est à l'origine des nombreux effets secondaires des chimiothérapies.
On sait que le développement de certains cancers est lié à la présence d'anomalies situées au niveau des gènes des cellules tumorales. Depuis une quinzaine d'années, des traitements ayant la capacité d'agir sur les conséquences de ces anomalies génétiques existent. Ces thérapies dites « ciblées » détruisent spécifiquement les cellules tumorales renfermant l'anomalie visée, entraînant potentiellement moins d'effets indésirables que la chimiothérapie.
Les progrès techniques en biologie moléculaire rendent désormais possible l'identification rapide de l'ensemble des anomalies présentes dans la tumeur d'un patient, à partir d'un fragment de celle-ci ou d'un prélèvement de sang (on parle de biopsie liquide qui permet de rechercher de potentielles anomalies à partir de l'ADN rejeté par les cellules cancéreuses dans le sang : l'ADN tumoral circulant). L'hypothèse actuellement privilégiée en cancérologie, basée sur l'expérience clinique acquise sur les thérapies ciblées, suggère qu'un meilleur contrôle de la maladie pourrait être obtenu en administrant à un patient la thérapie ciblée appropriée en fonction du profil moléculaire de sa tumeur (ciblant la/les anomalie(s) susceptible(s) d'être directement responsable(s) de son développement), plutôt qu'en se basant sur le type et la localisation de la tumeur pour le choix du traitement.
Cependant, certaines tumeurs ne présentent pas d'anomalie moléculaire spécifique pouvant être ciblée par un traitement, ou il n'existe pas encore de thérapie ciblée agissant sur les (l') anomalie(s) détectée(s).
Pour ces patients, les alternatives thérapeutiques en dehors de la chimiothérapie sont bien souvent limitées.

Plusieurs études cliniques récentes ont démontré l'efficacité de traitements stimulant le système immunitaire afin qu'il détruise les cellules cancéreuses. On parle d'*immunothérapie.* Depuis quelques années, plusieurs immunothérapies essentiellement des anticorps (tels que le Durvalumab et le Tremelimumab), ont été développées pour restaurer une réponse immunitaire efficace contre les cellules cancéreuses. Cependant, il est encore nécessaire d'améliorer l'utilisation de ces immunothérapies en augmentant leur efficacité, par exemple en testant leur association.

L'objectif principal de cette étude est d'évaluer si un traitement par immunothérapie (Durvalumab + Tremelimumab lors de la première administration de traitement puis Durvalumab seul pendant 48 semaines – soit 1 an de traitement) agissant sur deux voies différentes de stimulation du système immunitaire pourrait ralentir le développement des tumeurs.

2. LE TRAITEMENT ETUDIE

♦ Présentation des médicaments étudiés

Au moment où il vous propose cette étude, votre médecin investigateur connaît le profil moléculaire de votre tumeur. A ce jour, ce profil moléculaire ne permet pas de vous proposer un traitement par une thérapie ciblée. Un traitement par immunothérapie vous est donc proposé, afin d'aider votre organisme à restimuler votre système immunitaire pour lutter contre votre cancer.

Les deux médicaments utilisés dans cette étude (Durvalumab et Tremelimumab) sont des thérapies expérimentales ne disposant pas encore d'une autorisation de mise sur le marché par les autorités de santé européenne [Agence Européenne du Médicament (EMA)] ou française [Agence Nationale de Sécurité du Médicament et des produits de santé (ANSM)].
Ces deux immunothérapies, développées par le laboratoire Astra Zeneca, sont des anticorps monoclonaux humanisés capables de stimuler certaines cellules du système immunitaire pouvant détruire les cellules cancéreuses.

Durvalumab

Le **Durvalumab** sera administré à dose fixe de 1500 mg toutes les 4 semaines par perfusion intraveineuse à l'hôpital tant qu'un bénéfice sera observé ou jusqu'à progression de la maladie ou toxicité inacceptable.

Tremelimumab

Le **Tremelimumab** sera administré une seule fois à une dose de 300 mg par perfusion intraveineuse à l'hôpital.

♦ Quelles sont les alternatives médicales ?

Votre oncologue vous propose de participer à cet essai clinique, car il/elle considère qu'il s'agit de la meilleure approche thérapeutique dans votre cas. Cependant, il convient d'aborder avec votre oncologue les différentes alternatives thérapeutiques possibles. D'autres approches thérapeutiques peuvent être possibles en fonction de vos traitements antérieurs et de vos antécédents médicaux.

3. DEROULEMENT DE L'ETUDE

L'étude MOST *Plus* se déroulera sur une période estimée à 36 mois et votre participation durera tant qu'un bénéfice sera observé ou jusqu'à la progression de la maladie, ou toxicité inacceptable.

♦ Avant le début du traitement

Après signature du consentement de participation et avant de débuter le traitement, un bilan médical initial sera effectué afin de vérifier si vous répondez à tous les critères requis pour participer à cette recherche. Ce bilan médical comprendra les examens suivants :

- Un examen clinique complet (taille, poids), une mesure des signes vitaux (pouls, tension artérielle, température, rythme respiratoire), une revue de vos antécédents médicaux et de tous les traitements que vous prenez, une évaluation de votre état de santé général,
- Une prise de sang et une analyse d'urine,
- Un examen d'imagerie afin de préciser la taille et la localisation de votre tumeur,
- Un bilan cardiaque comprenant un électrocardiogramme (pour contrôler l'activité électrique de votre cœur),
- Un test de grossesse le cas échéant.

Si certains de ces examens ont été réalisés récemment, vous n'aurez pas forcément besoin de les réaliser à nouveau. Votre médecin investigateur avisera le cas échéant.

Si vous correspondez aux critères requis pour participer à cette recherche et que le bilan médical initial ne montre aucune contre-indication, vous pourrez recevoir le traitement de l'étude.

♦ Suivi au cours du traitement

Dès votre inclusion dans l'étude, vous serez traité(e) pendant 52 semaines (1 an) (« période d'induction ») avec de l'immunothérapie.

Différents cas de figure seront possibles suivant l'évolution de votre tumeur durant ces 52 premières semaines (1 an) de traitement :

- Si votre maladie progresse, cela signifie que le traitement est inefficace pour vous, il sera donc stoppé définitivement. Votre participation à l'étude sera terminée.

- Si votre maladie est stable ou si votre médecin investigateur constate une diminution de la taille de votre tumeur à la fin des 52 semaines, vous serez affecté(e) de manière aléatoire par tirage au sort (on parle de « randomisation ») à l'un des groupes suivants pour la deuxième partie de l'étude (« période d'entretien ») :

  - Groupe A (Maintien du Durvalumab) : Si vous êtes affecté(e) à ce groupe, vous continuerez à être traité(e) de manière continue par le Durvalumab seul, tant qu'un bénéfice sera observé.
  - Groupe B (Interruption du Durvalumab) : Si vous êtes affecté(e) à ce groupe, le traitement par Durvalumab sera interrompu. En cas de progression de votre maladie, votre médecin investigateur pourra vous traiter à nouveau selon la même séquence avec la combinaison thérapeutique Durvalumab + Tremelimumab (une injection) puis avec le Durvalumab seul (tant qu'un bénéfice sera observé ou jusqu'à progression de la maladie ou toxicité inacceptable) s'il le juge nécessaire.

Ni vous ni votre médecin investigateur ne pouvez choisir dans quel groupe vous serez affecté(e) : la randomisation est réalisée par un ordinateur et permet d'améliorer la qualité scientifique de l'étude.

Les différentes phases de l'étude et son déroulement sont schématisés ci-dessous :

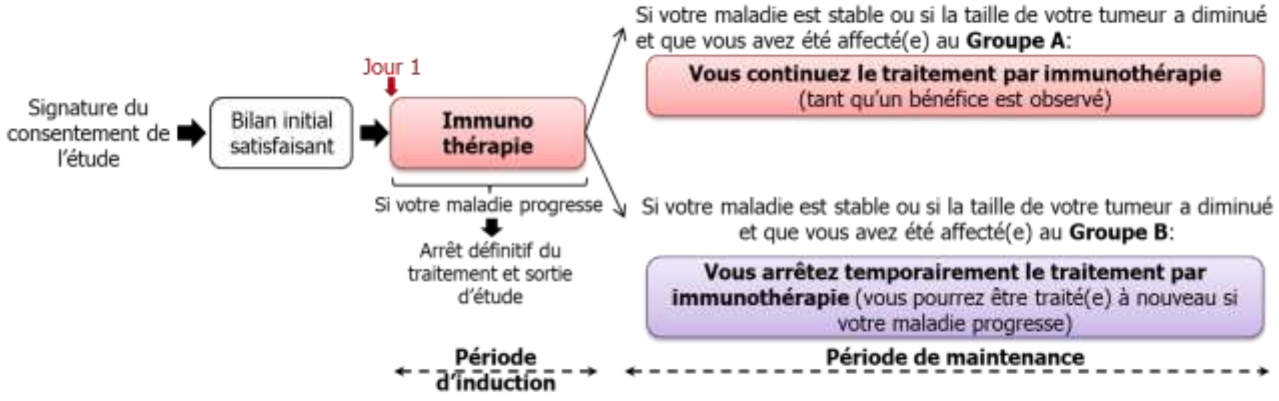

Au total, 25 patients seront affectés au groupe A et 25 patients au groupe B.

Calendrier des examens réalisés au cours de l'étude

Pendant toute la durée de votre participation à cette étude, des examens réguliers seront réalisés selon le calendrier présenté ci-dessous :

	**Période d'induction**					**Période d'entretien**	**Fin de traitement**
	Cycle 1			***Cycle 2***	***Cycle 3 à Cycle 13***	***Cycle 14 à Cycle X***	
	Jour 1	*Jour 7*	*Jour 15*	*Jour 1*	*Jour 1*	*Jour 1*	
**CYCLE DE TRAITEMENT**							
	Cycle 1, Jour 1 : Injection de Durvalumab et Tremelimumab puis Cycle 2 à Cycle 13, Jour 1 : Injection de Durvalumab seul					**Groupe A** : Cycle 14 à cycle X, Jour 1 : Injection de Durvalumab seul<br>**Groupe B** : Arrêt traitement à la fin du cycle 13 de traitement	
**EXAMENS**							
Examen clinique complet (dont poids, température et rythme respiratoire)	X	X	X	X*		X*	X
Pression artérielle et pouls	X*			X*	X*	X*	X
Imagerie médicale pour le suivi de votre tumeur					X : fin cycle 12 (semaine 48) et fin de cycle 13 (semaine 52)	Toutes les 8 semaines après la randomisation	X
Bilans sanguins	X		X	X	X	X	X
Analyse urinaire				X	X	X	X
Bilan cardiaque (ECG)	X			X	Si jugé nécessaire par le médecin		X
Recueil des évènements indésirables et des traitements concomitants	X						X
Questionnaire sur la qualité de vie						Au cycle 14 et 16 semaines après la randomisation	X

**Environ 30 minutes avant l'injection intraveineuse de Durvalumab, puis 30 minutes après le début de l'injection, et à la fin de l'injection.*

NB : Certains examens, dont des bilans sanguins additionnels, pourront être réalisés à la discrétion de l'investigateur s'il le juge pertinent d'un point de vue médical.

♦ Après le traitement

Une visite de fin de traitement sera réalisée soit à la fin de l'étude initialement prévue pour tous les participants soit avant la fin de l'étude si jamais vous désirez interrompre votre participation ou si votre médecin investigateur décide, dans votre intérêt, d'un arrêt définitif du traitement. Lors de cette visite, celui-ci discutera avec vous des modalités d'arrêt du traitement et de votre nouvelle prise en charge.
Après cette visite de fin de traitement deux autres visites de suivi à 1 mois d'intervalle sont prévues au cours desquelles vous aurez un examen physique, un bilan sanguin et urinaire. Ensuite vous serez revu tous les trois mois par votre médecin investigateur.
Afin de compléter les résultats de l'étude, votre médecin investigateur pourra être amené à se renseigner sur votre état de santé après votre sortie d'étude.

4. BENEFICES ATTENDUS, CONTRAINTES ET RISQUES PREVISIBLES

♦ Les bénéfices attendus

A ce jour, il n'est pas possible d'affirmer que votre participation à cette étude aura un bénéfice direct sur votre maladie mais le traitement proposé apparaît comme une option thérapeutique innovante. Les bénéfices potentiels peuvent être un arrêt de l'évolution de votre maladie voire une régression de votre tumeur.

Par ailleurs, les résultats issus de cette étude pourront également contribuer à l'avenir à améliorer la prise en charge d'autres patients atteints de cancer.

♦ Les contraintes : Que devrez-vous faire au cours de l'étude ?

✓ Être présent(e) aux visites prévues sur toute la durée de l'étude et suivre les indications données dans le cadre de l'étude.
  Les examens mentionnés dans le calendrier récapitulatif (voir ci-avant) sont des examens médicaux standards mais leur fréquence est plus élevée dans le cadre de cette étude notamment les bilans sanguins. Seules les biopsies tumorales sont des procédures spécifiquement réalisées dans le cadre de cette étude.

✓ Indiquer à votre médecin :
  - tous les traitements pris pendant toute la durée de votre participation à l'étude y compris ceux ne faisant pas suite à une prescription ainsi que les traitements homéopathiques et les remèdes naturels. Durant le traitement, certains médicaments vous seront interdits. Votre médecin vous en informera et vous indiquera si nécessaire par quoi les remplacer.
  - tous les effets et évènements indésirables (problèmes de santé en lien ou non avec la recherche), lésions, symptômes, y compris les éventuelles hospitalisations non planifiées.

  Pour cela, vous devrez remplir le document listé ci-dessous qui vous sera remis et le rapporter à chaque visite prévue dans le cadre de l'étude :
  - Un « carnet de surveillance à domicile » pour noter quotidiennement les événements, les anomalies ou les symptômes que vous pourriez constater durant l'étude ainsi que les éventuels médicaments pris pour les traiter. Ces indications seront très utiles à votre médecin pour apprécier votre tolérance au traitement.

✓ Conserver sur vous pendant toute la durée de votre participation à l'étude la « carte patient » qui vous sera remise. Elle vous identifie comme participant à cet essai clinique et indique un contact médical à joindre en cas d'urgence. Informez vos proches de son existence et présentez-la lors de toutes vos consultations chez un médecin.

✓ Si vous participez à cette recherche, vous ne pourrez pas participer simultanément à une autre recherche clinique (renseignez-vous auprès de votre médecin investigateur) pendant toute la durée de votre participation.

♦ Les risques prévisibles

Comme tous les médicaments, le Durvalumab et le Tremelimumab peuvent provoquer des effets indésirables, mais qui ne surviennent pas systématiquement chez tous les patients.
Les effets indésirables rapportés chez les patients ayant reçu la combinaison thérapeutique Durvalumab + Tremelimumab ou Durvalumab seul sont très similaires.

Fréquents (Au moins 10% des patients)

- Fatigue
- Nausées, Vomissements et Diarrhées, douleur abdominale
- Diminution de l'appétit,
- Démangeaison avec ou non éruption cutanée
- Difficultés respiratoires (essoufflement), Infections au niveau des voies respiratoires (dont pneumonie)
- Toux
- Fièvre
- Gonflement ou œdème (dû à l'accumulation de liquide)

- Dysfonctionnement de la thyroïde avec une diminution (hypothyroïdie) de la production des hormones thyroïdiennes qui peut se manifester par de la fatigue, une prise poids, une augmentation de la sensibilité au froid, une constipation, une faiblesse musculaire, un ralentissement du rythme cardiaque, des troubles de la mémoire, la peau sèche, le visage bouffi, des pertes des cheveux
- Inflammation du foie qui peut se manifester par un jaunissement de la peau ou des blancs des yeux, des urines foncées, des nausées importantes et des vomissements, des douleurs du côté droit du ventre, perte d'appétit, des démangeaisons au niveau de la peau, des saignements ou des bleus apparaissant plus facilement que d'habitude, une augmentation des taux sanguins de certaines enzymes du foie.

Moins Fréquents (Entre 1 et 10% des patients) :

- Inflammation des poumons qui peut se manifester par l'apparition ou l'aggravation d'une toux, des difficultés respiratoires accompagnées de fièvre, ou une pneumonie
- Dysfonctionnement de la thyroïde avec une augmentation (hyperthyroïdie) de la production des hormones thyroïdiennes qui peut se manifester par de l'anxiété ou de la nervosité, une perte de poids, un essoufflement, une sensation de chaleur et une augmentation du rythme cardiaque,
- Dysfonctionnement de la fonction rénale avec inflammation possible des reins,
- Altération du système nerveux central qui peut se manifester par une faiblesse inhabituelle des jambes, des bras et du visage, des sensations d'engourdissement et de fourmillement dans les mains ou les pieds. De rares cas d'inflammation sévères du système nerveux ont été observés qui peuvent entrainer une altération des cellules nerveuses ou de la connexion entre les cellules nerveuses et les muscles
- Il y a également un risque, faible mais possible, que vous développiez une réaction allergique au Durvalumab et/ou Tremelimumab (réaction au cours de l'injection IV ou juste après) qui peut chez certains patients s'avérer sévère. Ce type de réaction allergique se manifeste par une modification de la pression artérielle, des frissons, des difficultés respiratoires, des urticaires parfois sévères. Pour cette raison, vos signes vitaux seront suivis pendant et après l'injection de Durvalumab et de Tremelimumab,
- Inflammation du gros intestin qui peut se manifester par des douleurs abdominales et des diarrhées avec ou sans sang
- Altération de la fonction des surrénales à produire des hormones stéroïdiennes qui peut se manifester par des douleurs à l'estomac, des vomissements, une faiblesse musculaire, la fatigue, la dépression, une faible tension artérielle, une perte de poids, des problèmes rénaux et des changements d'humeur et/ou de personnalité. Cette altération peut devenir permanente et dans ce cas un traitement hormonal sera mis en place,
- Inflammation du pancréas qui peut se manifester par des douleurs abdominales, nausées, vomissements et un état de fatigue, et par une augmentation des enzymes pancréatiques (Amylase, Lipase)
- De plus, les patients atteints de différents types de cancer qui ont été traités avec Durvalumab seul ou en combinaison avec le Tremelimumab dans les essais cliniques ont couramment (c.-à-d. 1% à 10% des patients) rapporté: voix rauque, miction douloureuse, sueurs nocturnes, mycose buccale, douleur dans les muscles et les articulations.Syndrome grippal

Peu fréquents (Entre 0,01 et 1% des patients) :

- Problèmes intestinaux (colite) pouvant entraîner des déchirures ou des perforations de l'intestin (rares).
- Dysfonctionnement de l'hypophyse qui peut se manifester par des maux de tête, une sensation de soif, des troubles de la vision, des écoulements de lait maternel ou des cycles irréguliers chez les femmes
- Dysfonctionnement de la thyroïde se manifestant par une inflammation de la thyroïde.
- Apparition de bulles de grande taille sur des plaques rouges au niveau de la peau, souvent à l'origine de fortes démangeaisons (pemphigoïde).
- Infection dentaire ou des tissus mous de la cavité buccale
- Diabète de type 1 dit diabète insulinodépendant : Il s'agit d'une augmentation du taux de sucre dans le sang (hyperglycémie) qui peut se manifester par une prise de poids, une augmentation des urines, une augmentation de la faim et/ou de la soif. Ce type de diabète peut impliquer l'instauration d'un traitement par insuline

- Inflammation des muscles ou des tissus associés tels que les vaisseaux sanguins qui peut se manifester par une faiblesse, une sensation de fatigue en position debout ou lors de la marche, des douleurs musculaires persistantes

Rare (<0.1% des patients) :

- Inflammation du cœur qui peut se manifester par une douleur dans la poitrine et des troubles du rythme cardiaque (accélération ou au contraire ralentissement excessif)
- Inflammation de l'encéphale (encéphalite non infectieuse)
- Survenue d'un diabète insipide

De plus, les patients atteints de différents types de cancer qui ont été traités avec Durvalumab seul ou en combinaison avec le Tremelimumab dans les essais cliniques ont rarement (c.-à-d. moins de 0.1% des patients) rapporté : inflammation de la membrane entourant le cœur, augmentation de certaines cellules inflammatoires dans différentes parties du corps, inflammation de la couche intermédiaire de l'œil et d'autres événements impliquant l'œil (par exemple, l'inflammation de la cornée et les nerfs optiques), durcissement et le serrage de la peau et du tissu conjonctif, perte de la couleur de la peau, altération de certaines cellules du sang (globules rouges, plaquettes, neutrophiles), événements rhumatologiques (trouble inflammatoire causant une douleur ou rigidité musculaire et arthrite auto-immune), inflammation des vaisseaux sanguins (vascularite), inflammation des méninges (méningite non infectieuse) , infection de la vessie (cystite) immuno-induite, inflammation des voies biliaires dans le foie et en dehors du foie (cholangite sclérosante), réaction au niveau du site d'injection, embolie pulmonaire.

Remarque : Lors de l'administration du Durvalumab seul ou en combinaison avec le Tremelimumab, d'autres effets secondaires d'origine immunologique, qui n'ont pas encore été observés, peuvent survenir et peuvent se manifester une réaction inflammatoire dans n'importe quel organe ou tissu.

Les risques liés à la reproduction

Les effets du Durvalumab et du Tremelimumab sur les embryons et les fœtus ne sont pas connus. Toute grossesse (la vôtre ou celle de votre partenaire) survenant durant l'étude devra être immédiatement signalée à votre médecin.

Si vous êtes une femme : Il est interdit aux femmes enceintes de participer à cette étude et la participation des femmes en âge de procréer implique de :

- Réaliser un test sanguin de grossesse dans les 3 jours précédant le début du traitement
- Utiliser simultanément DEUX méthodes de contraception efficaces au cours de l'étude et **au moins jusqu'à 6 mois après l'arrêt du traitement**.

  Les méthodes de contraception listées ci-dessous sont considérées comme acceptables avec un risque d'échec de moins de 1%:

  -Dispositif intra-utérin : stérilet au cuivre, stérilet hormonal au Levonorgestrel (ex : Mirena®)

  -Méthodes hormonales : implants à l'etonogestrel (c'est-à-dire Norplan ou Implanon), injections au medroxyprogesterone (c'est-à-dire Depo-Provera), pilules contraceptives (combinées « normales » ou à « faible dose » et Cerazette), patch, anneau vaginal (c'est-à-dire ethinylestradiol et etonogestrel)

- Si vous avez des relations sexuelles sans méthodes de contraception ou si vous pensez être enceinte, vous devez IMMEDIATEMENT prévenir votre médecin.

L'allaitement, les dons de sang et d'ovocytes ne sont pas possibles durant la phase de traitement et jusqu'à 3 mois après l'arrêt du traitement de l'étude.

Si vous êtes un homme

Les hommes doivent accepter d'utiliser des préservatifs en cas de relation sexuelle **pendant toute la durée du traitement de l'étude et jusqu'à 6 mois après l'arrêt du traitement**. En outre, il est conseillé que votre partenaire féminine utilise un moyen de contraception très efficace pendant toute la durée de votre traitement et au cours du mois suivant l'arrêt de votre traitement. Si vous avez eu des relations sexuelles sans utiliser de préservatif ou si vous pensez que votre partenaire peut être enceinte, vous devez IMMEDIATEMENT prévenir votre médecin.

Les dons de sang et de sperme ne sont pas possibles pendant toute la durée de cette étude et jusqu'à 3 mois après l'arrêt des traitements de l'étude.

Eventuels effets non encore connus

Comme tout médicament, le Durvalumab et le Tremelimumab peuvent être à l'origine d'effets indésirables non encore identifiés. Pour votre sécurité, vous devrez signaler à votre médecin investigateur toute dégradation de votre état de santé, tout effet secondaire, toute prise de médicament et tout événement qui pourrait se produire, même si vous pensez qu'ils n'ont pas de rapport avec l'étude.

Les risques associés aux procédures de l'étude

Les **prises de sang** peuvent générer une certaine douleur et/ou rougeur au niveau du point de prélèvement.

Les **biopsies tumorales** exposent au risque de douleur lors du geste.
Le choix du site biopsié ainsi que les modalités du prélèvement seront adaptés à votre situation clinique de manière à réduire les désagréments et les risques potentiels. Une surveillance de quelques heures, ou une courte hospitalisation (24h maximum), après la réalisation du prélèvement pourra être nécessaire dans certains cas.
Des traitements antalgiques pourront être prescrits afin d'éviter ces évènements indésirables douloureux.
Ces procédures exposent également au risque théorique de saignement lors du geste ou dans les heures qui le suivent. Ce risque, bien que minime, devra être évoqué en cas de douleurs ou de fatigue dans les suites du geste.
Dans de rares cas, les biopsies peuvent entraîner des complications comme des problèmes pulmonaires (pneumothorax).

5. ECHANTILLONS BIOLOGIQUES PRELEVES DANS LE CADRE DE CET ESSAI CLINIQUE

Votre participation à cette recherche implique également le prélèvement d'échantillons biologiques (échantillons sanguins et échantillons tumoraux) **pour** des **analyses biologiques** dans le cadre de **l'étude biologique exploratoire associée à cet essai clinique.**

Ces analyses ont pour but de mieux comprendre l'action du traitement de l'étude et seront utiles à l'avancée des recherches scientifiques.

Les échantillons biologiques suivants seront prélevés selon le calendrier présenté ci-dessous :

TYPE	DETAILS
**OBLIGATOIRE**	
**ECHANTILLONS SANGUINS**<br>Environ 52mL à chaque temps de prélèvement	▪ **C1J1**<br>▪ **Fin de la période d'induction**<br>▪ **Au moment de la rechute de votre maladie**
**ECHANTILLON TUMORAL**<br>Biopsie tumorale si votre situation clinique le permet	▪ **Inclusion** (échantillon archivé ou nouvelle biopsie)<br>▪ **En cas de rechute de votre maladie (optionnel)**

6. VOTRE PARTICIPATION A CET ESSAI CLINIQUE : COMPLEMENT D'INFORMATION

Votre participation est volontaire, vous êtes libre d'accepter ou de refuser de participer à cette étude.
Pour pouvoir participer à cet essai clinique, vous devez être affilié(e) à un régime de sécurité sociale ou être bénéficiaire d'un tel régime.

♦ Dépenses et indemnisation

Votre participation à cette étude n'engendrera aucun frais personnel et ne donnera lieu à aucune indemnité financière. Les frais éventuels liés à l'étude seront pris en charge par le promoteur.

♦ Que se passera-t-il si vous ne souhaitez plus participer à l'étude, en cas d'arrêt prématuré de l'étude ou si vous en êtes exclu ?

Vous pouvez décider de mettre fin à votre participation à tout moment sans avoir à vous justifier. Cela ne changera en rien la qualité de la prise en charge et du suivi médical dont vous bénéficierez, ni vos relations avec l'équipe soignante.
Si vous décidez de quitter l'étude, les éléments biologiques et les données collectées jusqu'au retrait du consentement seront conservés et utilisés pour les besoins de la recherche.
Votre médecin investigateur garde la possibilité d'interrompre le traitement/votre participation à l'étude s'il juge que c'est dans votre intérêt, ou en cas de progression de votre maladie ou si vous ne pouvez plus suivre ses modalités.
Le promoteur ou l'autorité compétente peuvent également décider d'arrêter temporairement ou définitivement l'étude. Votre médecin investigateur vous informera alors de cette décision et il continuera à vous proposer les soins qui lui paraissent les plus adaptés à votre état de santé.

♦ Que se passera-t-il à la fin de cette étude ?

A la fin de votre participation à cette étude, votre médecin investigateur discutera avec vous des différentes options possibles pour votre prise en charge ultérieure.

♦ Qu'adviendra-t-il de mes échantillons biologiques ?

Il est possible que certains échantillons biologiques collectés ne soient pas intégralement utilisés dans le cadre de cette étude. Sauf opposition de votre part, ces échantillons seront conservés en France dans une structure agréée par le Ministère de la Recherche (Biobanque du Centre Léon Bérard) et pourront être utilisés à des fins de recherche scientifique et médicale sur votre maladie. Ces échantillons seront identifiés dans des conditions assurant leur confidentialité.
Vous pouvez vous opposer à cette utilisation ultérieure à tout moment et par tout moyen.

♦ Qu'adviendra-t-il de mes données personnelles ?

Dans le cadre de l'essai clinique MOST *Plus* auquel le promoteur vous propose de participer, un traitement de vos données médicales et personnelles va être mis en œuvre pour permettre d'analyser les résultats de la recherche au regard de l'objectif de cette dernière qui vous a été présenté. A cette fin, les données médicales et personnelles vous concernant seront transmises (par l'investigateur ou un membre habilité de son équipe) au promoteur de la recherche ou aux personnes ou sociétés agissant pour son compte dans des conditions assurant leur confidentialité. Ces données pourront également, dans des conditions assurant leur confidentialité, être transmises aux autorités de santé françaises ou étrangères. Les personnes chargées du contrôle de qualité de la recherche, dûment mandatées par le promoteur et sous la surveillance d'un professionnel de santé intervenant dans la recherche, auront accès aux données individuelles strictement nécessaires à ce contrôle. Elles sont soumises au secret professionnel.

Ces données, comme vos échantillons biologiques, seront identifiées par un numéro de code et vos initiales. **Seul le médecin qui vous suit dans le cadre de cette recherche connaît votre identité**. Vous pouvez y accéder, demander leur rectification ou vous opposer à leur transmission en vous adressant à lui/elle. Voir également ci-après *Quels sont vos droits en acceptant de participer à cette étude ?*

A l'issue de la recherche, elles seront archivées conformément à la réglementation en vigueur. **Ces données pourront être utilisées lors de recherches ultérieures exclusivement à des fins scientifiques**. En effet, l'utilisation de ces données permettra aux équipes de recherche de mieux comprendre les mécanismes et améliorer la prise en charge et le traitement des cancers. Vous pouvez retirer votre consentement à cette utilisation ultérieure ou d'exercer votre faculté d'opposition à tout moment.

♦ Quels sont vos droits en acceptant de participer à cette étude ?

Le **Promoteur** de cet essai ou étude clinique, qui en assure la gestion et la responsabilité, est le **Centre Léon Bérard** (Centre de Lutte contre le Cancer) situé 28 rue Laennec, 69373 LYON cedex 08 – France.

- Cette étude est réalisée conformément aux Bonnes Pratiques Cliniques (BPC), aux lois et aux réglementations européennes et françaises en vigueur concernant les recherches impliquant la personne humaine, notamment le Règlement (UE) n° 2016/679 Général sur la Protection des Données (RGPD).
  L'Autorité Compétente (ANSM) a autorisé cet essai le 28 juin 2013 sous le n° 130020A-12 RS. De plus, l'étude a reçu l'avis favorable d'un Comité de Protection des Personnes (CPP Sud-Est IV) en date du 15 janvier 2013.
- Conformément à la loi, le promoteur a souscrit une **assurance** garantissant sa responsabilité civile et celle de tout intervenant au cours de la recherche auprès de la société SHAM, 18 rue Edouard Rochet 69372 LYON Cedex 08 (police N° 142883). La garantie de cette assurance couvre les conséquences pécuniaires des sinistres trouvant leur cause génératrice dans cet essai, dès lors que la première réclamation est adressée à l'assuré ou à son assureur entre le début de la recherche et l'expiration d'un délai de dix ans courant à partir de la fin de celle-ci.
- **Votre consentement ne décharge en rien** l'investigateur et le promoteur de l'ensemble de leurs **responsabilités** et vous conservez tous vos droits garantis par la loi.
  Le promoteur est responsable du traitement des données collectées dans le cadre de cette recherche. Conformément aux lois et réglementations européennes et françaises en vigueur, dont le Règlement (EU) n° 2016/679 du 27 avril 2016 relatif à la protection des données personnelles et la loi n° 78-17 du 6 janvier 1978 relative à l'informatique, aux fichiers et aux libertés modifiée , vos données seront traitées dans le cadre de la recherche scientifique menée dans l'intérêt public dans le domaine de la santé (articles 6.1.e et 9.2.i du RGPD). Vos données pourront être transmises aux autorités de santé française dans des conditions assurant leur sécurité en particulier leur confidentialité, intégrité et disponibilité. Vos données ne seront pas transmises hors de l'Union Européenne.

  Vous disposez des droits suivants sur les données vous concernant :

  - **droit d'accès (Article 15),**
  - **droit de rectification des données (Article 16),**
  - **droit d'effacement des données (Article 17),**
  - **droit de limitation des données notamment si celui-ci venait à être remis en cause (Article 18).**

  Vous disposez également d'un **droit d'opposition** au traitement de vos données dans les conditions définies par le RGPD (Article 21).

  Ces droits s'exercent auprès du médecin qui vous suit dans le cadre de la recherche et qui seul connaît votre identité. Il pourra vous orienter vers le délégué à la protection des données du promoteur que vous pouvez contacter par mail à l'adresse suivante : dpd@lyon.unicancer.fr.

  Si malgré l'engagement du Centre Léon Bérard, à respecter vos droits et à protéger les données vous concernant, vous restez insatisfait, il vous est possible d'introduire une réclamation auprès de l'autorité de contrôle : la Commission nationale de l'informatique et des libertés (CNIL) en utilisant le lien : https://www.cnil.fr.

  Si vous retirez votre consentement, les données recueillies préalablement au retrait du consentement pourront ne pas être effacées et pourront continuer à être traitées dans les conditions prévues dans la recherche dans le cas où leur effacement compromettrait la réalisation des objectifs de l'étude (Article17).

  Vos données seront conservées pour un maximum de deux ans après la dernière publication scientifique liées au projet de recherche. Elles seront ensuite archivées pour un maximum de vingt-cinq ans.
- *Le promoteur atteste que le traitement informatisé des données personnelles recueillies dans le cadre de cette recherche sera réalisé conformément à la méthodologie de référence MR001 (relative aux recherches impliquant la personne humaine dans le domaine de la santé nécessitant le recueil du consentement de la personne concernée, consultable sur le site de la CNIL) ; la Direction de la Recherche Clinique du Centre Léon Bérard a effectué auprès de la Commission Nationale de l'Informatique et des Libertés (CNIL) une déclaration de conformité à cette méthodologie de référence, enregistrée sous le n°1994173 le 27/09/2016.*
- Vous pourrez être **informé(e) des résultats globaux** de la recherche une fois que celle-ci sera terminée en vous adressant à votre médecin investigateur.

- Tout au long de l'étude, vous pouvez **solliciter le médecin investigateur**, directement ou par l'intermédiaire d'un médecin de votre choix, afin d'obtenir **communication des informations qu'il détient concernant votre santé**.

Par ailleurs, votre médecin investigateur vous informera de toute information nouvelle susceptible d'avoir un impact sur votre santé, votre qualité de vie ou de remettre en question votre participation à l'étude.

7. A QUI S'ADRESSER EN CAS DE QUESTIONS OU DE PROBLEME ?

Vous pouvez vous adresser au Dr ....

qui pourra être contacté au numéro de téléphone suivant : ....

En son absence ou en cas d'urgence, vous pourrez contacter le Dr ....

au numéro de téléphone suivant : ....

CONSENTEMENT DE PARTICIPATION A L'ESSAI CLINIQUE

MOST *Plus*

Etude multicentrique de phase II en deux périodes, randomisée, en ouvert évaluant le bénéfice clinique d'un traitement d'entretien par une thérapie ciblée adaptée aux altérations moléculaires identifiées dans la tumeur pour des patients atteints de tous types de tumeurs solides localement avancées ou métastatiques en progression.

Version 6.0 du 14 juin 2022

Bras de traitement : Durvalumab + Tremelimumab

Promoteur : CENTRE LEON BERARD - 28 rue Laennec, 69373 LYON Cedex 08
Identifiants de la recherche : N° Promoteur : ET12-081 / N° EudraCT (base de données européenne) : 2012-004510-34

Je soussigné(e) : Nom :... Prénom : ....
Né(e) le ....
reconnais avoir été informé(e) par le Docteur..
de l'objet et des modalités du protocole de recherche **MOST *Plus.***

J'ai bien lu la note d'information qui m'a été remise explicitant l'objectif de l'étude, son déroulement, sa durée, ses contraintes, ses bénéfices et risques potentiels. J'ai pu poser toutes les questions que je souhaitais et j'ai reçu des réponses adaptées. J'ai bien compris les contraintes qui seront les miennes durant ma participation à cette étude et je les accepte. J'ai pu disposer d'un temps de réflexion suffisant entre l'information et la signature du présent consentement.

J'ai bien noté les coordonnées du médecin en charge de l'étude (et du médecin en cas d'urgence) que je pourrai contacter.

Je comprends que ma participation est volontaire et que je serai libre à tout moment d'y mettre fin sans avoir à en préciser les raisons et sans encourir aucune responsabilité ni aucun préjudice. Cela ne changera ni la qualité des soins que je recevrai ni ma relation avec l'équipe soignante.

En cas de retrait de mon consentement, les éléments biologiques déjà recueillis sur ma personne ainsi que les données personnelles collectées me concernant pourront être utilisés pour l'étude.

Je déclare sur l'honneur être affilié(e) à un régime de sécurité sociale ou bénéficiaire d'un tel régime.

J'accepte que les données qui me concernent fassent l'objet d'un traitement informatisé, par le promoteur ou pour son compte en France, afin d'analyser les résultats de la recherche au regard de l'objectif qui m'a été présenté. J'ai bien noté que toutes les données et informations qui me concernent resteront strictement confidentielles, traitées sans mention du nom et prénom, et ne seront consultées que par les organisateurs de cette étude, ou les personnes ou sociétés agissant pour son compte, et les représentants des autorités de santé. J'ai bien noté que mon identité n'apparaîtra dans aucun rapport ni publication. J'accepte que ces données soient utilisées lors de recherches ultérieures exclusivement à des fins scientifiques. Je peux retirer mon consentement à cette utilisation ultérieure ou exercer ma faculté d'opposition à tout moment.

J'ai été informé(e) que, conformément à la loi n°78-17 du 6 janvier 1978 modifiée et aux dispositions du Règlement (UE) n° 2016/679 relatif à la protection des données personnelles (RGPD), je dispose d'un droit d'accès de rectification, d'effacement et de limitation. Je dispose également d'un droit d'opposition à la transmission des données couvertes par le secret professionnel susceptibles d'être utilisées dans le cadre de cette recherche et d'être traitées.

J'ai été informé(e) de la possibilité d'accéder directement, ou par l'intermédiaire d'un médecin de mon choix, à l'ensemble de mes données personnelles et médicales en application des dispositions de l'article L.1111-7 du code de la santé publique.

Ces droits s'exercent auprès de mon médecin qui me suit dans le cadre de la recherche, qui seul connaît mon identité et qui contactera le promoteur de la recherche.

J'ai également été informé(e) que je peux m'adresser au médecin qui me suit ou au Délégué à la Protection des Données (DPD) du Centre Léon Bérard (dpd@lyon.unicancer.fr) pour obtenir des renseignements concernant la protection de mes données. Si malgré l'engagement du Centre Léon Bérard, à respecter mes droits et à protéger les données me concernant, je reste insatisfait, il m'est possible d'introduire une réclamation auprès de l'autorité de contrôle : la Commission nationale de l'informatique et des libertés (CNIL) en utilisant le lien : https://www.cnil.fr.

J'ai pris connaissance que le CPP Sud Est IV a donné un avis favorable à cette recherche le 15 janvier 2013 et que l'autorité compétente, l'Agence Nationale de Sécurité du Médicament et des produits de santé (ANSM), l'a autorisée le 28 juin 2013.

J'ai également été informé de la souscription par le promoteur d'une assurance garantissant sa responsabilité civile et celle de tout intervenant au cours de la recherche auprès de la société SHAM.

Mon consentement ne décharge en rien l'investigateur et le promoteur de la recherche de l'ensemble de leurs responsabilités et je conserve tous mes droits garantis par la loi.

Je pourrai à tout moment demander toute information complémentaire au médecin en charge de la recherche,

le Docteur ………………………………………………………………… Téléphone ………………………………………………

**Compte tenu des informations qui m'ont été transmises :**<br>*(cocher la case appropriée en fonction de votre volonté)*	**OUI**	**NON**
**J'accepte librement et volontairement de participer à cet essai clinique de me conformer aux procédures du protocole ainsi qu'aux prélèvements d'échantillons biologiques mentionnés comme obligatoires (sanguins et tumoraux).**	☐	☐
J'accepte librement et volontairement que les échantillons biologiques collectés dans le cadre de cette étude puissent être stockés et utilisés à des fins de recherche ultérieure.	☐	☐
Optionnel :<br>***Seulement pour les patients suivis au Centre Léon Bérard :***<br>J'accepte librement et volontairement que le prélèvement d'échantillon tumoral soit réalisé en cas de progression de ma maladie.	☐	☐

**Le patient**	**Le médecin investigateur**
Nom/Prénom :	Nom/Prénom :
Date :	Date :
Signature :	Signature :
